# The detection, characterization, and quantification of dominant degradation products of nisin A and Z in selected dairy products by liquid chromatography–high-resolution mass spectrometry technique

**DOI:** 10.3168/jdsc.2023-0392

**Published:** 2023-10-03

**Authors:** Sheena Wee, Sew Lay Chua, Dingyi Yu, Shoo Peng Koh, Kah Meng Lee, Yuansheng Wu, Sheot Harn Chan

**Affiliations:** 1 National Centre for Food Science, Singapore Food Agency, Singapore 609919; 2 Department of Food Science & Technology, National University of Singapore, Singapore 117543

## Abstract

Nisin, a bacteriocin produced through fermentation using bacterium *Lactococcus lactis*, has several commercial variants such as nisin A and nisin Z. Nisin serves as a natural preservative with antimicrobial properties in various food products, including dairy and beverages, for extending product shelf life. The efficacy and safety of nisin A as a bacteriocin has been well characterized. However, there is limited evidence regarding the efficacy, stability, and safety of nisin Z as a food preservative, as it has not undergone comprehensive regulatory reviews. In this work, we studied the stability of nisin A and Z in a selection of yogurt drinks and found nisin to be unstable, particularly in fruit-flavored yogurt drinks. Both nisin A and Z could experience significant degradation leading to the nisin parent ion peaks dropping below detectable level before the product's expiry date. Compared with nisin A, the formation of oxidized metabolite nisin Z+O appeared to be the predominant reaction for nisin Z. These findings highlight the need for further scientific research to understand the behavior of nisin Z under different application conditions, which is crucial for assessing the efficacy and safety of nisin Z under these conditions. One potential application of this knowledge is to optimize the formulation of yogurt-based drinks to stabilize nisin Z and sustain its biopreservative function throughout the product's shelf life. Additionally, the current study shows that for the testing of the presence of nisin A or nisin Z, it is imperative to cover both the parent and the main degradant(s) of nisin. This is especially true for nisin Z, for which the regulatory approval status may vary in different markets. As such, the confirmative identification of nisin Z and its key metabolites in commercial products would be essential.

## Highlights

• Fruit-flavored yogurt drinks promote formation of oxidized nisins as dominant degradants.
• Nisin Z appears to be more prone to oxidation than nisin A.
• Oxidation of nisin may potentially cause weakening or loss of its antimicrobial activity.
• This study highlights the need to stabilize nisin in food matrices to ensure its bioactivity.

## Graphical Abstract

**Figure:**
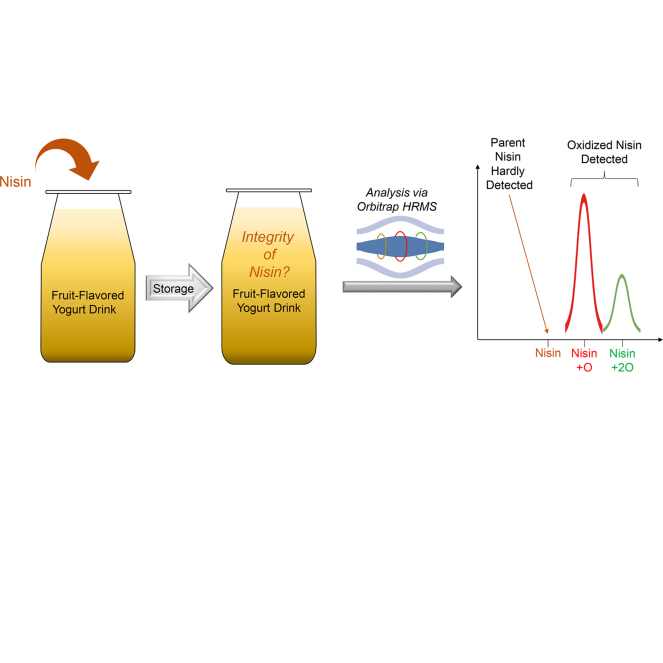
**Summary:** Nisin is a biopreservative widely used for dairy products. However, the stability of nisin in these food matrices appears to be not well established. Hence, it is uncertain that nisin can provide the expected efficacy throughout the product shelf life. We studied the stability of nisin A and Z in yogurt drinks by using orbitrap high-resolution mass spectrometry (HRMS). After a period of storage of the product, the nisin parent ion peak may drop below detectable levels, with nisin Z+O being the dominant form detected. This suggests that nisin degrades extensively, mainly via oxidation, in such products. This finding may help contribute to the understanding of the breakdown path of nisin Z and its impact on antimicrobial activity during the shelf life of the products. Our study results are also critical for performing robust regulatory reviews as well as food safety and quality control of processed foodstuffs that use nisin as a biopreservative.

Nisin, produced by *Lactococcus lactis* ssp. *lactis*, is a heat-stable bacteriocin and antimicrobial polypeptide. Comprising 34 amino acids, it has a molecular weight of 3.5 kDa (Hurst, 1981; Kaletta and Entian, 1989; Delves-Broughton et al., 1996). Due to its antimicrobial activity against gram-positive bacteria (Falahee et al., 1990; Thomas et al., 2000), nisin has been extensively used by food manufacturers as a biopreservative in various products such as dairy, meat, and beverages. It helps control foodborne pathogens and inhibit spoilage bacteria, thereby extending the shelf life of these products (Sobrino-López and Martín-Belloso, 2008).

Nisin has different variants, including nisin A and Z. The Joint FAO/WHO Expert Committee on Food Additives (JECFA), European Food Safety Authority (EFSA), and many other national food safety regulatory bodies have assessed nisin A to be a safe and effective preservative when it is added within regulatory limits to food and beverages (SCF, 1992; EFSA Panel on Food Additives and Nutrient Sources Added to Food et al., 2017). The US FDA granted nisin A with the status of generally recognized as safe (GRAS) in 1988. The Codex General Standard for Food Additives (GSFA Online, 2021) allows nisin A up to 12 mg/kg in flavored fluid milk drinks and dairy-based desserts, such as pudding, fruit, or flavored yogurt. Accurate quantification of nisin is hence essential to ensure prudent use of nisin within regulatory limits.

Nisin Z is a variant of nisin that was first reported by researchers in the Netherlands (Mulders et al., 1991). It differs from nisin A only by one amino acid with asparagine replacing histidine at position 27 (Matsusaki et al., 1998), rendering nisin Z more water soluble than nisin A. Being commercialized later in the 2000s, nisin Z appears to have been widely used in milk and dairy products in some countries. However, contrary to nisin A, there have been very few regulatory reviews conducted on the safety and efficacy of nisin Z. Consequently, there are uncertainties regarding the efficacy, stability, and safety of nisin Z used as a biopreservative for food. Therefore, it is necessary to initiate a study to examine the stability of nisin Z in food as an antimicrobial preservative to better understand the efficacy and safety characteristics of nisin Z.

According to Ibarra-Sánchez et al. (2020), nisin has been reported to lack stability in milk. Previous studies (Rollema et al., 1996, and Schneider et al., 2011) have identified truncation, hydration at residue 33, and oxidation (albeit to a limited extent) as the primary degradation products of nisin. Despite these degradation processes, the undegraded form of nisin, represented by the parent ion, has been found to be the most abundant. While hydration at residue 33 does not appear to alter the bioactivity of nisin, many other degradations, including oxidation, have been found to render nisin less bioactive (Rollema et al., 1996; Wilson-Stanford et al., 2009). Most of the nisin analysis methods by liquid chromatography-mass spectrometry (**LC-MS**) monitored only the parent ion (Ko et al., 2015; Lim et al., 2019). Considering the inherent instability of nisin in milk, monitoring the parent ion alone may not provide the full understanding of the breakdown path of nisin and its consequential effectiveness as an antimicrobial. Hence, it is desirable to quantify the dominant degradation products of nisin along with the parent.

Among the dairy products, yogurt drinks are believed to contain probiotics that aid lactose digestion (Savaiano, 2014), hence their popularity in the Far East and Southeast Asia region where 80% of the population is lactose intolerant (Sahi, 2009). However, to our best knowledge, there has been no existing study on the stability of nisin in this food category. In this work, the detection, characterization, and quantification of the dominant degradation products of nisin in fruit-flavored yogurt drinks were conducted using a LC high-resolution mass spectrometry (**HRMS**) system. We demonstrate that nisin is unstable in fruit-flavored yogurt drinks, to an extent that the nisin parent may become undetectable well before the designated expiry date of the yogurt drinks.

Formic acid (**FA**), trichloroacetic acid (**TCA**), and acetonitrile (**ACN**) were of LC-MS grade, reagent grade, and HPLC grade purchased from Fisher Scientific (Hampton, NH) and Sigma (St. Louis, MO). Analytical standards nisin A and Z were purchased from Sigma-Aldrich (St. Louis, MO; assay 2.5%) and Boc Sciences (Shirley, NY; assay 95%), respectively.

Nisin A and Z standards (100 mg/kg) in 20% ACN/0.5% FA were freshly prepared every day from the solid powder and further diluted to the relevant working concentrations.

The extraction protocol was adapted from Lim et al. (2019). Briefly, 0.45 mL of 4% TCA in ACN was added to 1 g of yogurt drinks and mixed well by vortexing for 15 s. Formic acid of 0.1% was then added to a final mixture volume of 5 mL, mixed well by vortexing for another 15 s and shaking on a horizontal shaker for 10 min at 260 rpm. This was followed by centrifugation of the mixture for 10 min at 4,006 × *g* at room temperature. The supernatant of 1 mL was then transferred to a 1.5-mL polypropylene centrifuge tube and centrifuged for another 10 min at 21,885 × *g* at room temperature. The supernatant of 15 µL was loaded onto LC-MS for analysis.

The processed sample supernatant of 15 µL was analyzed on a Dionex 3000 UltiMate LC (Thermo Fisher Scientific) coupled to a Q-Exactive Orbitrap HRMS (Thermo Fisher Scientific). The analytical column was a ZORBAX SB-Phenyl Solvent Saver Plus, 3.0 mm × 100 mm, 3.5 μm. The separation of the analytes was performed at 30°C with a flow rate of 0.6 mL/min using a 10 min gradient starting with 5% mobile phase B for the first 5 min, followed by a 2 min gradient ranging from 5% to 85% mobile phase B, then a 3 min gradient ranging from 85 to 90% mobile phase B, and subsequently a 1 min downward gradient ranging from 90 to 5% mobile phase B and maintained at 5% mobile phase B for 2 min. Mobile phase A is 10% ACN/0.5% FA and mobile phase B is 80% ACN/0.5% FA.

Full-scan MS spectra (*m*/*z* 150–2,000) was acquired under a mass resolution of 70,000, with a maximum ion injection time of 200 ms, and an automatic gain control (**AGC**) target of 1,000,000.

The targeted-MS2 scans were acquired in a separate run whenever necessary for characterization of targeted analytes. This was done with a resolution of 17,500, and an AGC target of 200,000, with a maximum injection time of 105 ms, multiplexed MS2 (**msx**) count of 1, isolation window of 1.6, collision energy of 17, and fixed first mass at *m*/*z* 100.0. The targeted precursor *m*/*z* were the third isotope, which is the isotope of highest intensity for each isotope cluster of nisin A, nisin Z, and their metabolites. The targeted precursor *m*/*z* were 671.7613, 674.9153, 678.1143, 667.1131, 670.3121, and 673.5111, corresponding to the third isotope of nisin A, nisin A+O, nisin A+2O, nisin Z, nisin Z+O, and nisin Z+2O.

The Skyline v21.2.0.425 (MacLean et al., 2010) software operating in molecule mode was employed to examine the isotope clusters. In cases where isotope(s) overlap with other co-eluting compound resulting in low isotope dot product (**IDP**), the isotope(s) will be excluded from area calculation. Isotope(s) used for area calculation are stated in the data sets appended (https://osf.io/g57k9/?view_only=65360cd817b644e88aa58f06bc815add).

Nisin A and Z were spiked into fruit-flavored yogurt drinks that did not contain nisin, extracted, and subjected to LC-MS analysis. The areas of parent ions were plotted against spiked nisin concentrations at 0 to 1,000 µg/kg.

To assess the recovery of nisin A and Z, 2 sets of spiked samples in fruit-flavored yogurt drinks were prepared: (1) nisin A and Z at 200, 600, and 1,000 µg/kg were spiked to blank fruit-flavored yogurt drinks that were tested to contain no nisin, then extracted as described earlier, which gave the pre-extraction spiked samples; and (2) the blank fruit-flavored yogurt sample was extracted as described, then nisin A and Z of 200, 600, and 1,000 µg/kg were spiked into the resulting blank extract, which gave the post-extraction spiked samples. The recoveries were calculated by comparing the peak areas of nisin A and Z of the pre-extraction spiked samples against their peak areas in the post-extraction spiked samples.

Nisin A and Z parents and their oxidized metabolites were extracted as described above. Freshly prepared standards at 300, 700, 1,000 µg/kg or 200, 600, 1,000 µg/kg were added for construction of calibration curve of nisin parents and their metabolites in standard addition mode. As nisin A and Z parents and their respective oxidized metabolites have similar chemical properties and hence presumably similar ionization efficiencies, the oxidized metabolites were quantified using the corrected standard addition curve of nisin parent. A similar pragmatic approach has previously been employed for quantification of oxidized methionine-containing peptides (Hammel et al., 2018; Silva and Erickson-Beltran, 2022). Only nisin parent or oxidized metabolites (or both) in milk- or yogurt-based beverages with Skyline IDP ≥0.9 were considered detected and further quantified.

To determine the presence of nisin A or Z following the product labels in our market survey testing, we initially attempted to detect and quantify nisin in fruit-flavored yogurt drinks according to the method reported by Ko et al. (2015). However, neither nisin A or Z was detectable by our LC-HRMS method. This led to our hypothesis that nisin added to these beverages had been fully degraded such that the nisin parent ion was no longer detectable. It thus triggered the current study because the ability to identify and quantify the degradation products in yogurt drinks was essential from both a regulatory and quality-control point of view.

We therefore performed simulation tests to search for the dominant metabolites of nisin A and Z in acidic solvent as a simple matrix and in yogurt drinks as real-world products. Nisin A and Z of 10 mg/kg were spiked into an acidic solvent consisting of 1.8% TCA and 45% ACN as well as into a fruit-flavored yogurt drink. The parents and the major degradation products were monitored on d 0, 3, 7, and 10.

The characterization of nisin degradation products was performed by manual examination of the MS1 spectra of the isotope clusters obtained from averaging the extracted ion chromatograms of the potential nisin degradation products and by manual examination of their MS2 spectra. The IDP processed using Skyline software were also employed to examine the isotope clusters. The Skyline IDP is an indicator of how close the detected isotope clusters (the first 5 isotopes having the highest intensities) match the theoretical isotope clusters. Only detected nisin isotope clusters with IDP ≥0.9 were accepted as good matches with the theoretical isotope clusters.

The results of the simulation test are shown in Figure 1. Nisin A and Z showed a difference in their degradation products progressively. In d 0, only nisin A and nisin A+H_2_O isotope clusters were observed in the acidic solvent and in yogurt drink spiked with nisin A. This was confirmed by IDP of 0.97 to 0.99 and accurate masses (the experimental masses of the 5 most intense isotopes within 6 ppm of the theoretical masses; data set 1a-b). Meanwhile, an isotope cluster of nisin Z and an isotope cluster consisting of the overlap of nisin Z+H_2_O and nisin Z+O were detected in both acidic solvent and yogurt drinks spiked with nisin Z in d 0. As the number of days progressed, the intensity of the degradation products relative to the parents increased. For nisin A in acidic solvent, only hydrated nisin A (singly and doubly hydrated nisin A) were observed at d 0, 3, 7, and 10, with nisin A+2H_2_O detected from d 7. In the fruit-flavored yogurt drink, an isotope cluster consisting of nisin A+O and nisin A+H_2_O was observed only on d 3. On d 10, the isotope cluster of degradation products seems to consist of nisin A+O only (IDP = 0.95). Accurate masses (data set 1a-b) and targeted MS2 spectra of nisin A+O in yogurt drink on d 10 in comparison to MS2 spectra of nisin A in acidic solvent and yogurt drink on d 0 and 10 (data set 1c) further corroborated the identity of nisin A+O.

**Figure 1:**
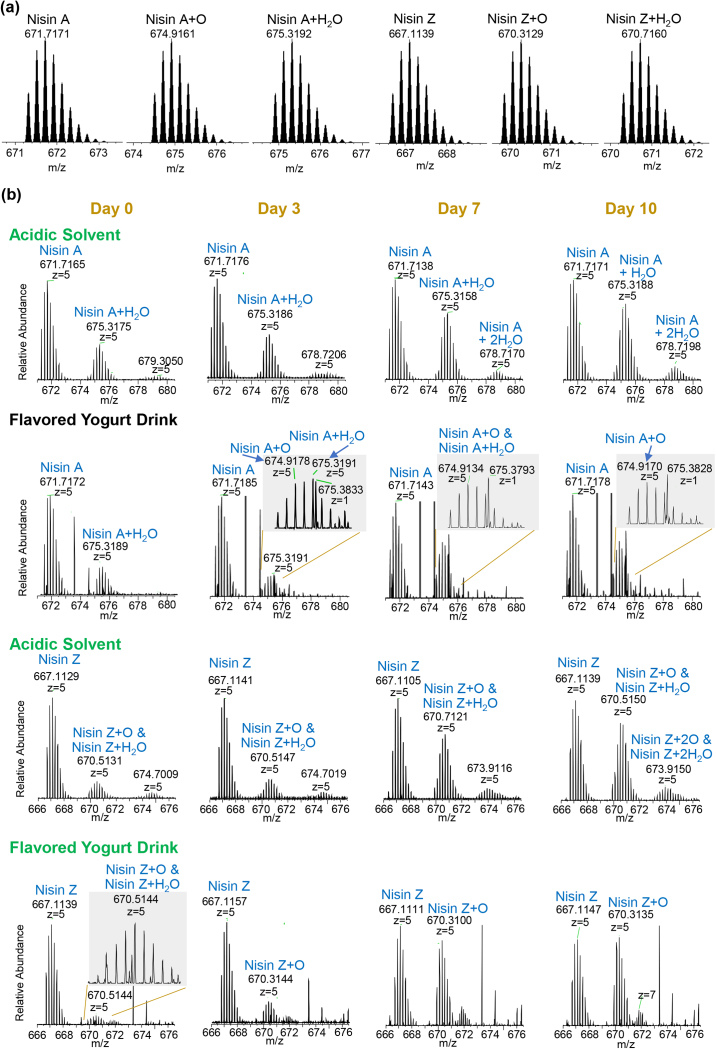
(a) Theoretical isotope clusters of nisin A, nisin A+O, nisin A+H_2_O, nisin Z, nisin Z+O, nisin Z+H_2_O; (b) actual observation of nisin A and Z and their respective metabolites from the simulation tests, in which nisin A and Z were spiked into an acidic solvent and into flavored yogurt drink with their major degradation products monitored on d 0 to 10.

On the contrary, in acidic solvent spiked with nisin Z, an isotope cluster consisting of nisin Z+O and nisin Z+H_2_O was observed from d 0 to 10, suggesting nisin Z exhibits a significantly stronger tendency for oxidation as compared with nisin A. From d 7, an isotope cluster consists of nisin Z+2O and nisin Z+2H_2_O was also detected. In contrast, in fruit-flavored yogurt drinks, deducing from the isotope patterns, the degradation product of nisin Z detected from d 7 onward seemed to consist only of nisin Z+O. The IDP of 0.99 and accurate masses (data set 1a-b) confirmed this. Targeted MS2 spectra of nisin Z+O in yogurt drink on d 10 in comparison to MS2 spectra of nisin Z in acidic solvent and yogurt drink on d 0 and 10 (data set 1c) further corroborated the identity of nisin Z+O. The MS1 intensity of nisin Z+O was similar to that of nisin Z on d 10. We postulate that as the number of days increases, the intensity of nisin Z+O may exceed the intensity of nisin Z, and nisin Z+2O may also be detected. The intensities of the dominant degradants of nisin A and Z relative to the parent molecules in acidic solvents and yogurt drinks on d 0 to 10 are shown in Figure 2. The slight increase of total intensity of nisin Z parent and dominant degradants from d 0 and 3 in acidic solvent (plot “b” of Figure 2) is assessed to be within experimental variation.

**Figure 2:**
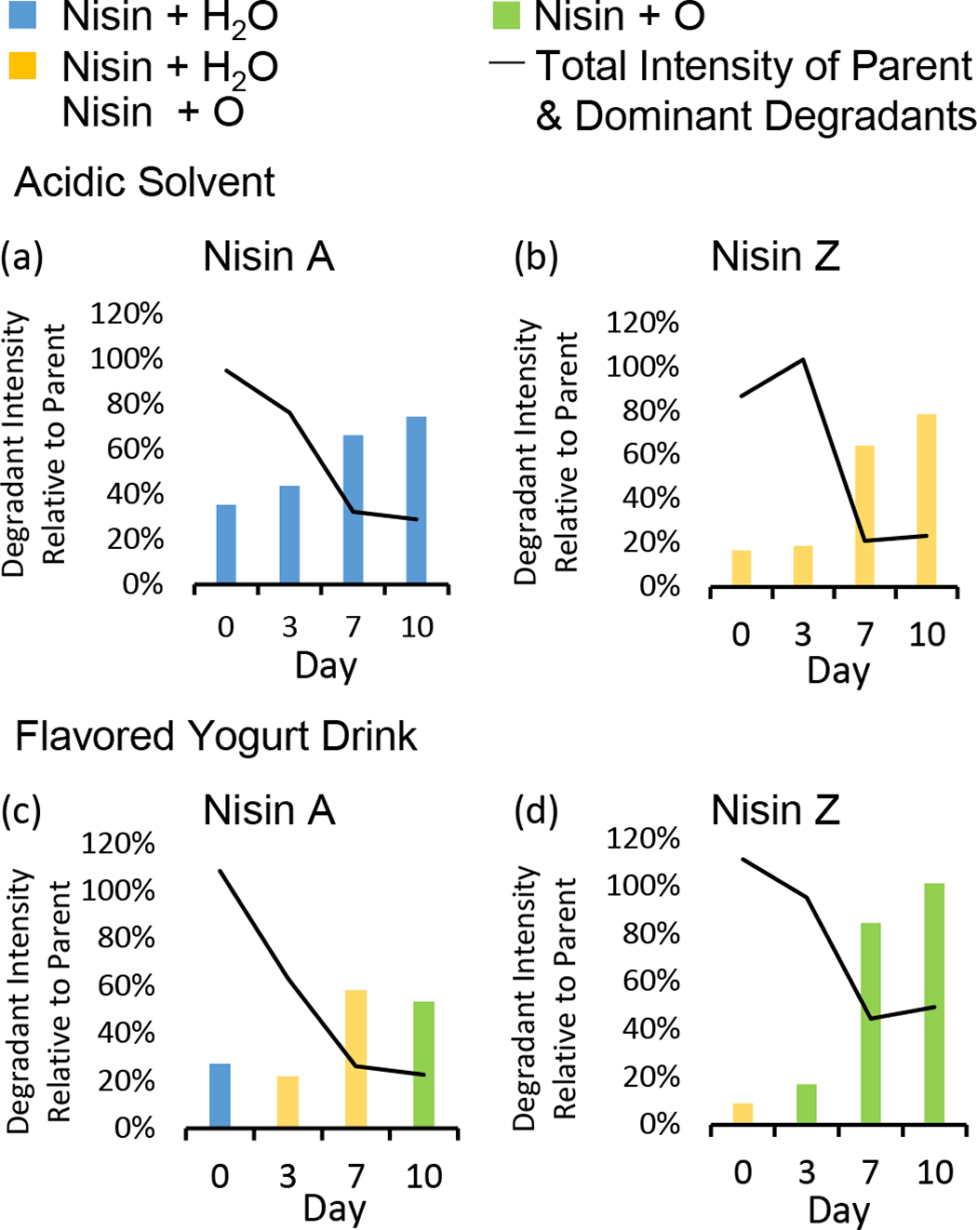
Intensities of the dominant degradants of nisin A and Z relative to the parents (100%) as well as total intensity of parent and dominant degradants observed in acidic solvent and yogurt drinks on d 0 to 10.

These results show that fruit-flavored yogurt drinks promote oxidation of nisin A and Z such that the oxidized metabolite becomes the dominant form of nisin in the above food matrices. Examination of the structures of nisin A and Z revealed that nisin has 7 sulfurs prone to oxidation on the side chains of methionines, lantionines, and β-methyllanthionines. In fruit-flavored yogurt drinks with various ingredients such as proteins, fats, and flavoring substances, nisin may adopt a different conformation from that in the acidic solvent, minimizing hydration reaction yet exposing more oxidizable sites for oxidation. As oxidation of nisin may potentially cause it to lose its antimicrobial activity (Rollema et al., 1996, and Wilson-Stanford et al., 2009), having appropriately formulated products to stabilize nisin A and Z in food matrices would be of paramount importance to ensure its bioactivity (Ibarra-Sánchez et al., 2020).

Nisin Z appears to be more prone to oxidation compared with nisin A based on our study. Being more hydrophilic than nisin A (Matsusaki et al., 1998), nisin Z is likely to adopt a slightly different conformation from nisin A, with nisin Z having more exposed oxidizable sites for oxidation to occur. Being a variant of nisin, which has been subjected to less intensive regulatory review, more studies are needed to understand the stability and degradation pathways of nisin Z in various food matrices. Exploration of various product formulations to increase the stability of nisin, in particular nisin Z, is essential to ensure the antimicrobial activity of nisin is preserved throughout the entire shelf life of the food products.

Nisin A and Z were spiked into fruit-flavored yogurt drink from 50 to 1,000 µg/kg, extracted, and subjected to LC-MS analysis. The concentration range of nisin A and Z with number of data points ≥8 and IDP ≥0.9 was found to be 200–1,000 µg/kg (data set 2a-b). The peak areas of nisin A and Z parent were plotted against this concentration range and the curves were found to be linear with R^2^ > 0.99. Limits of detection and quantification calculated from these curves were 60 µg/kg (round up to the nearest 10 µg/kg) and 200 µg/kg (round up to the nearest 100 µg/kg), respectively. Extraction recovery of nisin at 200, 600, and 1,000 µg/kg was found to be >85% (data set 2c).

We conducted an analysis of nisin parents and their oxidized metabolites in 10 different yogurt drink products from various sources. Source A claimed their yogurt drinks were specially formulated to exclude the use of nisin as a biopreservative before exporting to Singapore. Our tests confirmed the above claim (i.e., neither nisin A or nisin Z, nor their metabolites, was detected). The rest of the products from source B–D listed nisin in their ingredient lists. Our study revealed the presence of nisin Z in the form of its parent compound, oxidized metabolites, or both. The HRMS data characterized by Skyline IDP for isotope pattern fitting and accurate masses were presented in the form of the MS2 spectra of the dominant metabolites in the individual samples in data set 3. Among the 7 yogurt drinks, 6 appeared to have been supplemented with nisin Z. Notably, the degraded metabolites were dominated by oxidized metabolite nisin Z+O. In sharp contrast to the fruit-flavored yogurt drinks, the oxidized metabolite nisin Z+O was absent in the nonflavored (original) yogurt drink, suggesting that the fruit flavoring substances in the yogurt drinks promote oxidation of nisin Z. The quantification results of nisin Z and its oxidized metabolites in the yogurt drinks are shown in Table 1 and data set 3.

**Table 1:** Amount and form of nisin Z and metabolites detected from the yogurt drinks surveyed

Sample	Type of flavor in the yogurt drink surveyed	Source	Level detected (μg/kg)			Sum of nisin Z parent and oxidized metabolites (μg/kg)
			Nisin Z	Nisin Z+O	Nisin Z+2O	
1	Apple	A	—1	—	—	—
2	Strawberry	A	—	—	—	—
3	Original	A	—	—	—	—
4	Vanilla ice cream	B	—	562	—	562
5	Strawberry	B	—	742	—	742
6	Fruit	B	—	505	268	773
7	Apple	C	—	910	—	910
8	Coconut	C	—	560	268	828
9	Original	D	319	—	—	319
10	Strawberry	D	—	529	—	529

1 — = below the limit of quantification.

Using LC-HRMS and Skyline software, we found nisin A and Z to be unstable in fruit-flavored yogurt drinks. Oxidized metabolites were the major degradation products for nisin Z, in particular. Nisin Z+O rather than nisin Z was the dominant form detected in the commercial fruit-flavored yogurt drinks. As exemplified by the present work on the study of the dominant degradants of nisin Z in yogurt drinks, only with in-depth information about the degradation behavior of nisin Z and its impact on the antimicrobial activity after degradation can there be a solid foundation for the development of optimized product formulation to ensure lasting efficacy throughout the intended shelf life and for seeking regulatory approval for the usage scope and level of nisin Z in commercial products. Additionally, our study highlights the importance of analyzing both nisin A and Z, along with their dominant metabolites, in commercial products. This analysis is necessary to ensure compliance with food labeling regulations and permitted maximum levels. By considering all these factors, sound scientific judgments can be made and informed regulatory decisions can be taken.
